# Atrial and Ventricular Involvement in Acute Myocarditis Patients with Preserved Ejection Fraction: A Single-Center Cardiovascular Magnetic Resonance Study

**DOI:** 10.3390/jcdd11070191

**Published:** 2024-06-25

**Authors:** Riccardo Cau, Francesco Pisu, Giuseppe Muscogiuri, Jasjit S. Suri, Roberta Montisci, Luca Saba

**Affiliations:** 1 Department of Radiology, Azienda Ospedaliero Universitaria (A.O.U.), di Cagliari—Polo di Monserrato s.s. 554 Monserrato (Cagliari), 09045 Cagliari, Italy; fra.pisu1@gmail.com; 2 School of Medicine and Surgery, University of Milano-Bicocca, 20126 Milan, Italy; g.muscogiuri@gmail.com; 3 Department of Radiology, IRCCS Istituto Auxologico Italiano, San Luca Hospital, 20149 Milan, Italy; 4 Stroke Monitoring and Diagnostic Division, AtheroPoint™, Roseville, CA 95661, USA; jsuri@comcast.net; 5 Department of Cardiology, Azienda Ospedaliero Universitaria (A.O.U.), di Cagliari—Polo di Monserrato s.s. 554 Monserrato (Cagliari), 09045 Cagliari, Italy; rmontisci@unica.it

**Keywords:** myocarditis, atrial strain, ventricular strain, preserved ejection fraction

## Abstract

Cardiac magnetic resonance (CMR) is commonly employed to confirm the diagnosis of acute myocarditis (AM). However, the impact of atrial and ventricular function in AM patients with preserved ejection fraction (EF) deserves further investigation. Therefore, the aim of this study was to explore the incremental diagnostic value of combining atrial and strain functions using CMR in patients with AM and preserved EF. This retrospective study collected CMR scans of 126 consecutive patients with AM (meeting the Lake Louise criteria) and with preserved EF, as well as 52 age- and sex-matched control subjects. Left atrial (LA) and left ventricular (LV) strain functions were assessed using conventional cine-SSFP sequences. In patients with AM and preserved EF, impaired ventricular and atrial strain functions were observed compared to control subjects. These impairments remained significant even in multivariable analysis. The combined model of atrial and ventricular functions proved to be the most effective in distinguishing AM patients with preserved ejection fraction from control subjects, achieving an area under the curve of 0.77 and showing a significant improvement in the likelihood ratio. These findings suggest that a combined analysis of both atrial and ventricular functions may improve the diagnostic accuracy for patients with AM and preserved EF.

## 1. Introduction

Acute myocarditis (AM) is an inflammatory disease of the myocardium with different clinical presentations and outcomes [1,2,3,4]. The heterogeneous clinical presentation makes the diagnosis challenging, mainly in patients with preserved ejection fraction (EF), in which the disease may go undetected (EF) [5,6]. However, the ITAMY study demonstrated that even patients with myocarditis and preserved EF developed major adverse cardiac events with an incidence of about 8% [6]. In this scenario, cardiovascular magnetic resonance (CMR) is the diagnostic tool of choice for patients with suspected myocarditis, allowing for an accurate morphological and functional cardiac assessment [2,7,8,9,10]. CMR has shown to be useful for the evaluation of myocardial strain, enabling the assessment of subclinical systolic and diastolic impairment [9,11]. Among strain acquisition techniques, CMR feature tracking (CMR-FT) is an emerging non-contrast quantitative method to evaluate both atrial and ventricular myocardial deformation using routinely acquired cine-CMR images [8,9,11,12,13,14]. This method utilizes optical flow to identify features within the image and subsequently tracks them through successive images in the sequences [11]. Myocardial strain measures the rate of myocardial deformation between relaxed and contracted states during a cardiac cycle [11,15,16,17]. The myocardium is composed of different layers with varied orientations of myofibers in each layer. Longitudinal strain indicates the shortening of the subendocardial fibers along the longitudinal axis from the base to the apex, and it is denoted by negative values. Circumferential strain measures the concentric myocardial shortening of subepicardial fibers on a short axis-view, and it is expressed by a negative value. Radial strain evaluates myocardial thickening and thinning of both subepicardial and subendocardial in the radial direction towards the center of the LV, and it is represented by a positive value [11,15,16]. Due to the distinctive orientations and thinness of the atrial wall, typically only the longitudinal strain is measured at the atrial level. Left atrial (LA) function can be divided into three consecutive phases, namely (1) reservoir, reflecting atrial filling during systole; (2) conduit, a measurement of the passive left atrium emptying during ventricular diastole; and (3) booster, representing atrial contractility [9]. Several studies investigated the impact of atrial and ventricular functions in patients with AM, reporting atrial and ventricular dysfunction during the acute phase of AM [18,19,20]. Conversely, there is a paucity of data with controversial results on CMR-FT in patients with AM patients and preserved EF. Gatti et al. demonstrated no differences in global longitudinal (GLS), global circumferential strain (GCS), and global radial strain (GRS) of the LV between AM patients with preserved EF and healthy subjects [21]. On the other hand, Meindl et al. reported a significant reduction in LV myocardial strain function compared to control subjects [22]. An echocardiography study of 30 patients with acute myocarditis and preserved EF, confirmed by CMR according to the Lake Louise criteria, demonstrated atrial impairment over LV dysfunction [23]. The aim of this study was to evaluate the impact of atrial and ventricular strain dysfunction and the benefit of multi-chamber investigation over the course of acute myocarditis with preserved EF.

## 2. Materials and Methods

### 2.1. Study Population

In this retrospective, longitudinal, observational, single-center study, all consecutive patients with acute myocarditis who underwent CMR and fulfilled the modified Lake Louise Criteria between 3 March 2019 and 7 August 2022 were included. Clinically suspected AM was defined based on the recent European Society of Cardiology position paper [2]. In particular, symptomatic patients with chest pain who fulfilled at least one diagnostic criterion (new electrocardiogram modification, elevated troponin, wall motion abnormalities with preserved LVEF on echocardiography) were considered to have suspected AM. A definitive diagnosis of AM was established using the current Lake Louise Criteria via CMR [7]. Endomyocardial biopsy (EMB) was not performed in our hospital in this low-risk population according to international statement [2]. Ejection fraction was assessed via CMR in each subject. In total, 52 healthy age- and gender-matched subjects were recruited to serve as a control group.

Exclusion criteria included subjects <18 years old; patients with reduced LVEF; previous myocardial infarction; pre-existing cardiomyopathy; and suspected or known prior irreversible myocardial damage.

The Institutional Review Board’s approval for this retrospective, cross-sectional study was obtained, and patients’ consent was waived because of the retrospective nature of this study.

**Figure 1:**
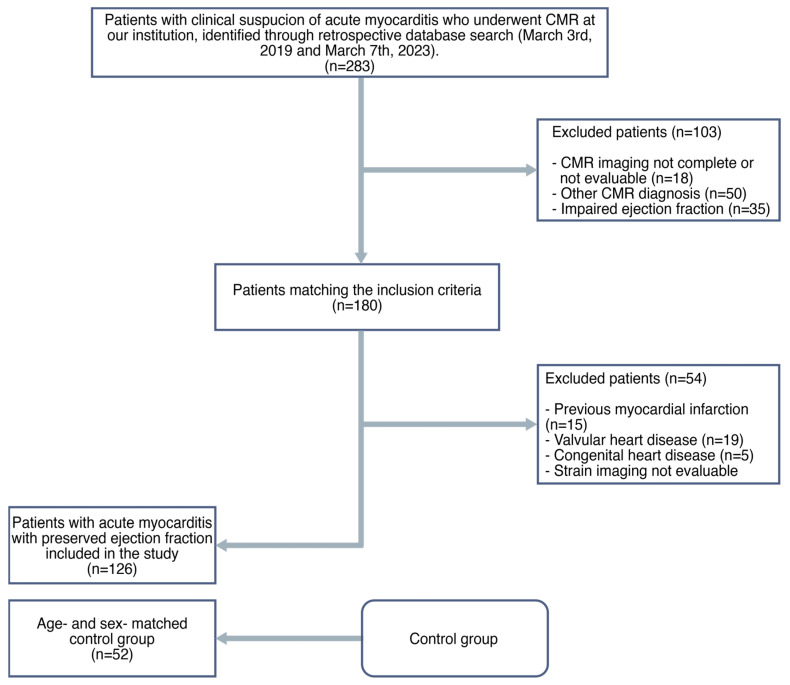
Flowchart of this study.

A flowchart demonstrating the application of inclusion and exclusion criteria is provided in Figure 1.

### 2.2. CMR Acquisition

CMR scans were performed at 4.1 ± 2.6 days (median = 1 day, range = 1–10 days) after admission to the hospital by using a Philips Achieva dStream 1.5 T scanner system (Philips Healthcare, Best, The Netherlands). Anterior coil arrays were used. All cine-images were acquired using a balanced steady-state free precession and retrospective gating during expiratory breath-hold maneuvers (TE: 1.7 ms; TR: 3.4 ms/flip-angle: 45°, section thickness = 8 mm) in both long-axis (two-, three- and four-chamber view) as well as short-axis planes with whole ventricular coverage from base to apex. 

T2 mapping was acquired before the administration of contrast-media on three representative short-axis slices (at the base, mid-ventricular, and apex, respectively) using a single-breath-hold, black-blood prepared ECG-triggered, spin-echo multiecho sequence.

Late gadolinium enhancement (LGE) imaging was performed in both long- and short-axis slices 10–12 min minutes after contrast media injection (Gadovist, Bayer Healthcare, Berlin, Germany) with a dose of 0.15 mL per kg body weight using phase-sensitive inversion recovery sequences (PSIR) (TE: 2.0 ms; TR: 3.4 ms; flip-angle: 20°, section thickness = 8 mm) with an inversion time determined using the Look–Locker technique.

### 2.3. CMR Image Post-Processing

We used the commercially available software system Circle CVI42 (CV42 6.0, CVI42, Circle Cardiovascular Imaging Inc., Calgary, AB, Canada) for CMR-FT data analysis. Offline CMR feature tracking analyses were conducted for evaluation of peak global longitudinal strain, global radial strain, and global circumferential strain in a 16-segment software-generated 2D model. Concerning longitudinal strain, data on myocardial strain were derived from two-, three-, and four-chamber long-axis views. Regarding radial strain and circumferential strain, data on myocardial strain were derived from apical, mid-ventricular, and basal short-axis views in all patients. On all images, the epi- and endocardial borders were traced in end-diastole. After that, an automatic computation was triggered, by which the applied software algorithm automatically outlined the border throughout the cardiac cycle. 

Similarly, CMR-FT analyses of atrial deformation were conducted offline. On all the acquired images, LA endocardial borders were manually traced in long view of the cine-images when the atrium was at its minimum volume. In particular, the four-, three-, and two-chamber views were used to derive LA longitudinal strain. LA appendage and pulmonary veins were excluded from segmentation. 

**Figure 2:**
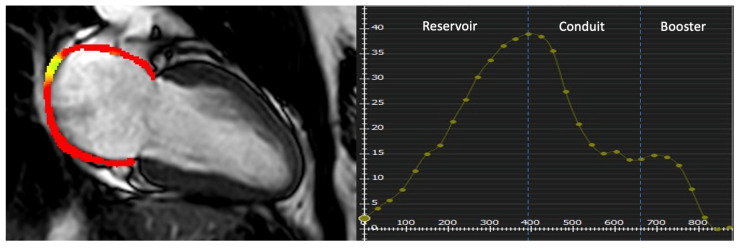
Examples of LA strain parameters in patient with AM. The endo- and epicardial borders of the LA were manually depicted, and the curves of the LA function were automatically obtained. Corresponding LA reservoir, conduit, and booster strain curves are shown.

After that, with an automatic computation, the software algorithm automatically tracked the myocardial borders throughout the cardiac cycle. The quality of the tracking and contouring was visually validated and manually corrected when needed. There are three peaks in the strain curve, including reservoir, conduit, and booster strain, as shown in Figure 2. 

Accordingly, their corresponding strain rate parameters were included. The quality of the tracking and contouring of atrial and ventricular function was visually validated and manually corrected by a radiologist with 4 years of experience in cardiac imaging. 

The extent and location of LGE were assessed using both qualitative and quantitative methods. Qualitatively, the evaluation involved counting and determining the location of affected myocardial segments. Quantitatively, the extent of LGE was measured by tracing the epicardial and endocardial contours in each short-axis image. A region of interest (ROI) was manually placed in the myocardium without LGE to serve as a reference. LGE was defined as myocardium with a mean signal intensity exceeding the reference ROI by more than 5 standard deviations.

### 2.4. Statistical Analysis

Continuous variables are presented as mean ± standard deviation. Comparisons of continuous data were performed using the independent samples *t*-test or Mann–Whitney U test; Kolmogorov–Smirnov tests were used to assess the normality of residuals. Categorical variables were compared by using the chi-squared test or Fisher’s exact test, as appropriate. 

The association of LA and LV function parameters with the presence of AM with preserved EF was assessed by using univariable and multivariable logistic regression analysis. The univariable regression models included the presence of AM as the dependent variable and each LA and LV strain parameter as an independent variable. A subset of CMR parameters that showed a *p* value < 0.05 in univariable analysis was further examined in multivariable analysis. All multivariable models were adjusted for age, sex, and LVEF.

To investigate the incremental value of considering both atrial and ventricular involvement, we compared multivariable models using the log-likelihood ratio (LR) test. Concretely, we compared models of ventricular or atrial parameters considered alone (base models) with models that included both parameters (complex models). The diagnostic accuracy of statistically significant parameters was further evaluated using receiver operating characteristics analysis. Confidence intervals (CIs) around the median area under the curve (AUC) were computed with 5000 bootstrap iterations.

A *p* value < 0.05 was considered statistically significant. All statistical analyses were performed using IBM SPSS Statistics version 22 (SPSS Inc., Chicago, IL, USA) and R (R Foundation for Statistical Computing, Vienna, Austria, version 4.1.0).

## 3. Results

### 3.1. Patient Demographics, Clinical Data, and CMR Data

Table 1 shows the characteristics of this study population. A total of 126 patients with AM and preserved EF, consisting of males (99, 78%) and females (27, 21%) with a mean age of 44.72 ± 18.22 years, as well as 52 age- and sex-matched control subjects, were included. There was no difference in the cardiovascular risk profile between the groups under study, except for some participants having a family history of coronary artery diseases (*p* = 0.001). 

The median time from symptom onset to CMR was 5 days (interquartile range 2–7 days)**.** Ejection fraction was preserved in the myocarditis group as well as in the control group (56.48% ± 5.99 vs. ± 60.52% ± 5.54, *p* = 0.008, Table 1). No differences were found in LV volumes and sizes quantified according to CMR imaging between the AM and control subjects.

Regarding LV myocardial strain parameters, GLS (−13.60% ± 3.25 vs. −16.03 ± 2.35, *p* = 0.001), GCS (−14.60% ± 3.91 vs. −17.69 ± 3.27, *p* = 0.001), and GRS (22.48% ± 9.00 vs. ± 30.23 ± 8.08, *p* = 0.001) demonstrated impaired function between the AM group and control subjects.

The LA reservoir mechanism was reduced in patients with myocarditis compared to control subjects (31.21 ± 11.88 vs. 36.09 ± 7.68, *p* = 0.001). Also, the LA conduit function was impaired in the myocardium compared to the control group (17.87 ± 9.77 vs. 21.61 ± 6.52, *p* = 0.004). In contrast, there was no difference in the LA reservoir strain rate, LA conduit strain rate, and contractile booster pump function between the groups under analysis. 

### 3.2. Association of LA and LV Strain Mechanism with AM

Table 2 summarizes the findings of univariable and multivariable logistic regression analyses, which were conducted to examine the association between the AM, LA, and LV strain parameters. There was a significant association between the reservoir phase, conduit phase, and the presence of AM (odds ratio [OR] = 1.043, 95% CI 1.010–1.076, *p* = 0.009 and OR = 1.048, 95% CI 1.009–1.089, *p* = 0.001; respectively). In the model adjusted for traditional cardiovascular risk factors and LVEF, the association remained significant (OR = 1.044, 95% CI 1.006–1.083, *p* = 0.021 and OR = 1.069, 95% CI 1.016–1.125, *p* = 0.010; respectively). 

Moreover, there was a significant association between GLS (OR = 0.738, 95% CI 0.642–0.849, *p* = 0.001), GCS (OR = 0.776, 95% CI 0.692–0.869, *p* = 0.001), GRS (OR = 1.117, 95% CI 1.065–1.171, *p* = 0.001), and the presence of AM. In the fully adjusted model, the association remained significant (GLS, OR = 0.726, 95% CI 0.611–0.842, *p* = 0.001; GCS OR = 0. 801, 95% CI 0.699–0.917, *p* = 0.001; GRS OR = 1.104, 95% CI 1.042–1.170, *p* = 0.001; respectively). Association of LVEF as a primary factor with AM was also statistically significant.

### 3.3. Added Value of LA and LV Strain Parameters in Combination for Diagnosing AM

The diagnostic performance of LA and LV strain parameters alone versus their integrated models were evaluated using the LR test. The results showed that integrating LV and LA strain parameters resulted in a systematically better diagnostic accuracy than either parameter alone (Table 3). The integration of GLS, GCS, and GRS with the conduit resulted in a better discrimination ability (LR 31.73, 34.42 and 35.91, respectively) than the conduit (LR 23.37) diagnosing AM alone (*p* = 0.004, *p* < 0.001 and *p* < 0.001, respectively). Including GLS, GCS, and GRS in addition to the reservoir significantly improved the discrimination ability for diagnosing AM (LR 30.95, 32.70 and 34.73, respectively, vs. LR 21.96 for reservoir alone; *p* = 0.003, *p* = 0.001 and *p* < 0.001, respectively). The diagnostic performance of LVEF alone compared to the integrated models of LVEF and atrial and ventricular strain parameters is shown in Supplementary Table S1. 

**Figure 3:**
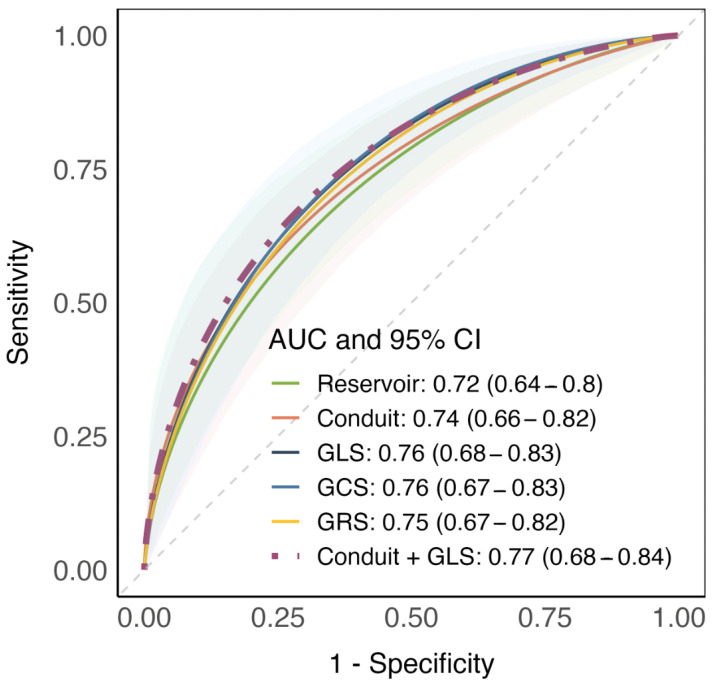
Receiver operating characteristics analysis reporting diagnostic performance of logistic regression models using atrial and ventricular strain measurements. The best performing model is reported with a violet, dashed line. The median area under curve and 95% confidence interval are reported for each model. Abbreviations are the same as in Table 1.

In ROC analysis, the model that integrated GLS with the conduit strain outperformed both the LA and LV strain parameters in diagnosing AM, as indicated by the higher AUC (0.77, 95% CI 0.68–0.84, Figure 3). All other combined models are reported in Supplementary Figures S1 and S2. The ROC analysis for LVEF models is presented in Supplementary Figure S3. LVEF models have lower AUCs compared to models of conduit and global ventricular strains.

## 4. Discussion

Our data support the hypothesis that atrial and ventricular strain impairment is present in AM patients despite a preserved EF, suggesting subtle systolic and diastolic impairment. The main results of the current study can be summarized as follows: (1) a subtle ventricular impairment of all three myocardial layers was present despite normal EF, (2) besides ventricular dysfunction, the LA reservoir and conduit are significantly reduced, and (3) a multi-chamber approach improves the diagnostic accuracy, providing insights into the physiological communication between LV cardiac chambers in AM.

Previous studies evaluated the ventricular mechanism in AM patients with preserved EF with controversial results [20,21,24]. We found alterations of longitudinal, circumferential, and radial strain parameters in comparison with previous studies that reported no significant differences or involvement of longitudinal myocardial fiber alone during the acute stage of AM with preserved EF. These differences may be explained by the cut-off values used for normal EF. In particular, we decided to use a cut-off value for normal EF of 50% according to the 2022 AHA/ACC/HFSA Guideline for the Management of Heart Failure [25]. In addition, we included only a selected group of AM patients who fulfilled the modified Lake Louise Criteria [7].

Studies comparing left atrial mechanisms in patients with myocarditis have demonstrated reservoir and conduit impairment with preserved booster pump mechanisms in AM patients [19,20]. LA is an active cardiac chamber that plays a central role in cardiac output through an interaction with LV across the entire cardiac cycle [9,13]. 

However, little is known about the impact of the LA mechanism in AM patients with preserved EF. Given the relationship between LA dysfunction and adverse outcomes in various cardiovascular diseases [9,26,27], identifying atrial impairment in patients with preserved EF may aid in risk stratification. Meindl et al. investigated the diagnostic value of LV and LA myocardial strains in AM patients with preserved EF using speckle tracking echocardiography, demonstrating reservoir and conduit dysfunction in patients with acute myocarditis compared to healthy controls [23]. Similarly, Lee et al. showed impaired reservoir and conduit mechanisms in AM patients using CMR [28]. Our data confirmed these results, supporting the hypothesis of abnormalities in atrial compliance and diastolic impairment throughout the course of AM, despite preserved EF.

Overall, our results suggest abnormalities in both atrial and ventricular mechanisms in AM patients, emphasizing the physiological “communication” between left cardiac chambers during the course of the disease. Indeed, LA strain parameters depend on LV systolic function, especially subendocardial fibers, which can be evaluated by LV GLS [29].

Inoue et al. investigated LA strains as markers of LV diastolic impairment in a multicenter cohort of 322 patients with cardiovascular disease of different etiologies, reporting that the strongest determinant of LA strain parameters was LV GLS [30]. In addition, the authors demonstrated that the correlation between LA strain and LV diastolic dysfunction was more limited in patients with preserved EF (AUC of 0.67) or preserved GLS (AUC of 0.58) compared to those with impaired EF (AUC of 0.80) [30]. Similar results were also reported by Carluccio et al., supporting the significant contribution of LV impairment to LA dysfunction in patients with impaired EF [31].

Given this intrinsic relationship between LA and LV chambers, the LA strain may be a sub-optimal stand-alone marker of LV diastolic dysfunction in patients with normal EF [29], highlighting the importance of a comprehensive approach that includes both atrial and ventricular strain parameters. In particular, combining GLS and conduit mechanisms enhanced the diagnostic performance of CMR in AM patients compared to using atrial and ventricular strain parameters alone.

### 4.1. Clinical Implications

Our data emphasize the importance of evaluating multi-chamber strain parameters as a supportive non-contrast CMR diagnostic feature in AM patients, even with preserved EF. The integration of these parameters does not require additional CMR sequences and can be routinely acquired in standard CMR protocols, requiring only short post-processing examinations. This approach may be particularly helpful for patients who would benefit from abbreviated CMR protocols due to the inability to undergo lengthy CMR examinations or contrast media administrations. Confirming these results in a larger cohort of AM patients may facilitate a more in-depth understanding of multi-chamber involvement in AM patients with preserved EF. Indeed, the assessment of atrial and ventricular strains could potentially carry significant clinical implications, indicating that AM patients with preserved EF and impaired atrial and ventricular strains may constitute a distinct subgroup with subtle systolic and diastolic dysfunction. This finding ideally encourages therapeutic approaches to be applied earlier, potentially leading to improved outcomes for these patients.

### 4.2. Limitations

The current study’s limitations should be acknowledged. Firstly, the relatively small sample size and the retrospective nature of this study are limitations. However, we exclusively enrolled very homogenous AM patients with preserved EF. The promising results of our study could prompt further prospective trials, including a larger number of patients, to confirm our findings. 

Second, AM is more common in men. This prevalence of AM may influence the analysis of myocardial strain. To avoid bias, we matched the two groups under analysis for age and sex and used a multivariable regression model that included age and sex to account for their potential confounding effects. Although we observed significant differences in LVEF between these groups in both univariable and multivariable analyses, LVEF demonstrated lower AUC values compared to models incorporating conduit and global ventricular strains. This highlights the need for future studies to incorporate a wider range of variables to account for potential confounding factors and to provide a more robust understanding of the relationship between myocardial strain and LVEF in AM patients. Third, there was no systematic endomyocardial biopsy to detect acute myocarditis. Finally, this study was cross-sectional in design, and we did not assess the predictive value of myocardial strain parameters for adverse cardiovascular events in AM patients, nor did we examine the data on patients’ treatments during follow-up. 

In our single-center study, we evaluated AM patients with the same CMR scanner and protocol, which may limit the reproducibility of our results. Externally validated multi-center longitudinal studies are needed to assess the association of these CMR parameters with patient outcomes.

## 5. Conclusions

Besides the dysfunction of ventricular strain parameters, patients with AM and preserved EF demonstrated impaired reservoir and conduit mechanisms. By combining atrial and ventricular mechanisms, CMR-FT can serve as a useful tool for assessing cardiac function and for gaining insights into multi-chamber dysfunction in the context of AM.

## Figures and Tables

**Table 1:** Baseline characteristics and CMR parameters of included patients. The data are presented as mean ± standard deviation (SD) for quantitative variables and n (%) for qualitative variables.

	AM Subjects with Preserved EF (n = 126)	Control Subjects (n = 52)	*p* Value
Age, y	44.72 ± 18.22	46.73 ± 15.38	0.490
Male, n (%)	99 (78%)	43 (82%)	0.536
Weight, kg	71.53 ± 12.53	70.38 ± 17.31	0.778
Height, cm	170.70 ± 6.73	165.07 ± 10.57	0.136
BSA, m^2^	1.82 ± 0.17	1.83 ± 0.16	0.187
Hypertension, n (%)	22 (17%)	11 (21%)	0.567
Dyslipidemia, n (%)	13 (10%)	6 (11%)	0.727
Obesity, n (%)	15 (12%)	3 (6%)	0.255
Smoke, n (%)	19 (15%)	8 (16%)	0.857
Diabetes, n (%)	5 (4%)	3 (6%)	0.559
Family history of CAD, n (%)	22 (17%)	1 (2%)	**0.001**
Elevated troponin, n (%)	126 (100%)	/	/
LVEF, %	56.48 ± 5.99	60.52 ± 5.54	**0.008**
LVEDV/BSA, mL/m^2^	83.07 ± 15.15	80.37 ± 17.25	0.322
LVESV/BSA, mL/m^2^	34.59 ± 13.23	32.22 ± 9.06	0.119
LVSV/BSA, mL/m^2^	50.27 ± 10.15	48.18 ± 9.47	0.226
LV mass/BSA, g/m^2^	62.87 ± 15.57	60.10 ± 10.70	0.216
LGE presence, n (%)	121 (96%)	/	/
LGE extent, %	7.81 ± 4.23	/	/
Reservoir, %	31.21 ± 11.88	36.09 ± 7.68	**0.001**
Reservoir rate, s^−1^	1.42 ± 0,58	1.46 ± 0.42	0.597
Conduit, %	17.87 ± 9.77	21.61 ± 6.52	**0.004**
Conduit rate, s^−1^	−1.91 ± 1.00	−1.73 ± 0.60	0.214
Booster, %	13.22 ± 4.99	13.52 ± 4.17	0.711
Booster rate, s^−1^	−1.69 ± 0.60	−1.82 ± 0.62	0.218
GLS, %	−13.60 ± 3.25	−16.03 ± 2.35	0.001
GCS, %	−14,60 ± 3.91	−17.69 ± 3.27	0.001
GRS, %	22.48 ± 9.00	30.23 ± 8.08	0.001

Abbreviations: AM, acute myocarditis; CAD, coronary artery disease, EDV, end-diastolic volume; ESV, end-systolic volume; SV, stroke volume; EF, ejection fraction; BSA, body surface area; GLS, global longitudinal strain; GCS, global circumferential strain; GRS, global radial strain. Bold indicates statistical significance.

**Table 2:** Univariable and multivariable logistic regression analysis of CMR variables for discrimination between AM patients and controls. Each multivariable model was adjusted for age, sex, and left ventricle ejection fraction. Multivariable model for LVEF was adjusted for age and sex. Abbreviations are the same as in Table 1.

	Univariable Analysis		Multivariable Analysis	
	OR (95% CI)	*p* Value	OR (95% CI)	*p* Value
Reservoir, %	1.043 (1.010–1.076)	0.009	1.044 (1.006–1.083)	0.021
Conduit, %	1.048 (1.009–1.089)	0.001	1.069 (1.016–1.125)	0.010
GLS	0.738 (0.642–0.849)	0.001	0.726 (0.611–0.842)	0.001
GCS	0.776 (0.692–0.869)	0.001	0. 801 (0.699–0.917)	0.001
GRS	1.117 (1.065–1.171)	0.001	1.104 (1.042–1.170)	0.001
LVEF	0.918 (0.870–0.966)	0.001	0.921 (0.872–0.968)	0.002

**Table 3:** Incremental value of combining ventricular and atrial strain measurements to identifying AM patients with preserved ejection fraction. All multivariable logistic regression models include age, sex, and left ventricle ejection fraction. Base models of conduit and reservoir strain are updated with one atrial strain measurement at a time, and these updated models are compared with the corresponding base model using a Likelihood ratio test. LR indicates log-likelihood ratio; Df, degrees of freedom. Other abbreviations are the same as in Table 1.

	Adjusted R^2 a^	LR	Δ LR	Df	*p* Value ^b^
Conduit ^c^	0.175	23.37		5	<0.001
+GLS	0.233	31.73	8.36	6	0.004
+GCS	0.251	34.42	11.05	6	<0.001
+GRS	0.261	35.91	12.54	6	<0.001
Reservoir ^c^	0.166	21.96		5	<0.001
+GLS	0.228	30.95	8.99	6	0.003
+GCS	0.239	32.70	10.74	6	0.001
+GRS	0.252	34.73	12.77	6	<0.001

^a^ McFadden pseudo R^2^. ^b^ *p* value by Likelihood ratio test. ^c^ base models of conduit and reservoir strains are compared with the null model.

## Data Availability

The data underlying this article cannot be shared publicly due to the privacy of the individuals that participated in this study. The data may be shared upon reasonable request from the corresponding author.

## Supplementary Materials

The following supporting information can be downloaded at: https://www.mdpi.com/article/10.3390/jcdd11070191/s1, Supplementary Figure S1. Receiver operating characteristics analysis reporting diagnostic performance of logistic regression models using conduit and global ventricular strain measurements; Supplementary Figure S2. Receiver operating characteristics analysis reporting diagnostic performance of logistic regression models using reservoir and global ventricular strain measurements. Supplementary Figure S3. Receiver operating characteristics analysis reporting diagnostic performance of logistic regression models using left ventricle ejection fraction and atrial and global ventricular strain measurements. The best performing model is reported with a dark blue, dashed line. The median area under curve and 95% confidence interval are reported for each model. Supplementary Table S1. Incremental value of combining left ventricle ejection fraction (LVEF, primary factor) with ventricular and atrial strain measurements in identifying acute myocarditis patients with preserved ejection fraction. All multivariable logistic regression models include age and sex as adjustment factors. Base models of LVEF are updated with one atrial and ventricular strain measurement at a time, and these updated models are compared with the corresponding base model using a Likelihood ratio test. LR indicates log-likelihood ratio; Df, degrees of freedom. Other abbreviations are the same in Table 1 in the main text.

## Abbreviations

AM, acute myocarditis; CMR, cardiovascular magnetic resonance; CMR-FT, cardiovascular magnetic resonance feature tracking; EF, ejection fraction; GCS, global circumferential strain; GLS, global longitudinal strain; GRS, global radial strain; LGE, late gadolinium enhancement; LR, log-likelihood ratio; LV, left ventricle; ROC, receiver operating characteristics; RV, right ventricle.
