# Supplementary material for: Atrial and Ventricular Involvement in Acute Myocarditis Patients with Preserved Ejection Fraction: A Single-Center Cardiovascular Magnetic Resonance Study
Source: J Cardiovasc Dev Dis. 2024 Jun 25;11(7):191. doi: 10.3390/jcdd11070191 (PMC11276940; doi:10.3390/jcdd11070191)
Supplement: Supplementary file 1 [file jcdd-11-00191-s001.zip › jcdd-3035583-supplementary.pdf]

Atrial and ventricular involvement in acute myocarditis patients with preserved ejection fraction: A single-center cardiovascular magnetic resonance study.

Content	Page number
Supplemental Figure Legends	2
Supplemental Figure S1. Receiver-operating characteristics analysis reporting diagnostic performance of logistic regression models using conduit and global ventricular strain measurements.	3
Supplemental Figure S2. Receiver-operating characteristics analysis reporting diagnostic performance of logistic regression models using reservoir and global ventricular strain measurements.	4

Supplemental Tables

Supplemental Table S1. Incremental value of combining left ventricle ejection fraction (LVEF, primary factor) with ventricular and atrial strain measurements in identifying acute myocarditis patients with preserved ejection fraction. All multivariable logistic regression models include age and sex as adjustment factors. Base model of LVEF are updated with one atrial and ventricular strain measurement at a time, and those updated models are compared with the corresponding base model using a Likelihood ratio test. LR indicates log-likelihood ratio; Df, degrees of freedom. Other abbreviations as in Table 1 in the main text.

	Adjusted R2[a]	LR	Δ LR	Df	P value[b]
LVEF	0.125	16.36		4	< 0.001
+Conduit	0.172	22.90	6.54	5	< 0.001
+Reservoir	0.162	21.55	5.19	5	< 0.001
+GLS	0.222	30.15	13.80	5	< 0.001
+GCS	0.232	31.64	15.29	5	< 0.001
+GRS	0.221	32.02	13.66	5	< 0.001

Supplemental Figure Legends

Supplemental Figure S1. Receiver-operating characteristics analysis reporting diagnostic performance of logistic regression models using conduit and global ventricular strain measurements. The best performing model is reported with an orange, dashed line. The median area-under-curve and 95% confidence interval is reported for each model. GLS indicated global longitudinal strain; GCS, global circumferential strain and GRS, global radial strain.

Supplemental Figure S2. Receiver-operating characteristics analysis reporting diagnostic performance of logistic regression models using reservoir and global ventricular strain measurements. The best performing model is reported with a dark blue, dashed line. The median area-under-curve and 95% confidence interval is reported for each model. Abbreviations as in Supplementary Figure S1.

Supplemental Figure S3. Receiver-operating characteristics analysis reporting diagnostic performance of logistic regression models using left ventricle ejection fraction, atrial and global ventricular strain measurements. The best performing model is reported with a dark blue, dashed line. The median area-under-curve and 95% confidence interval is reported for each model. Abbreviations as in Supplementary Figure S1.

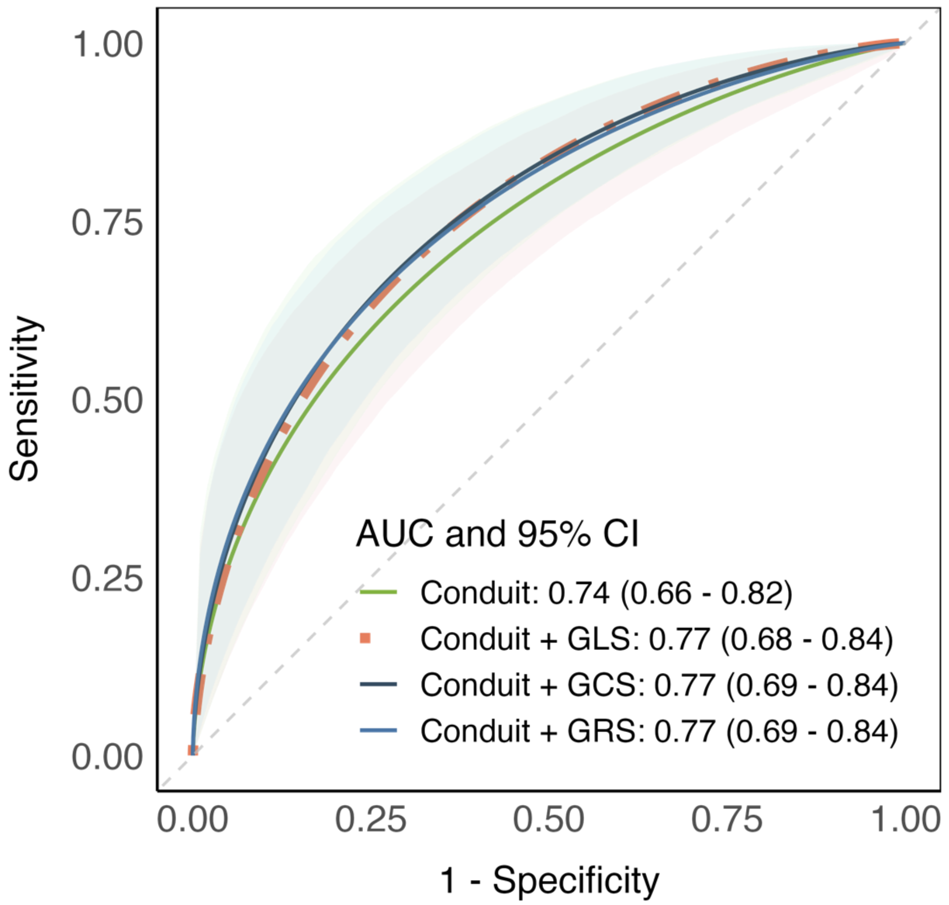

Supplemental Figure S1.

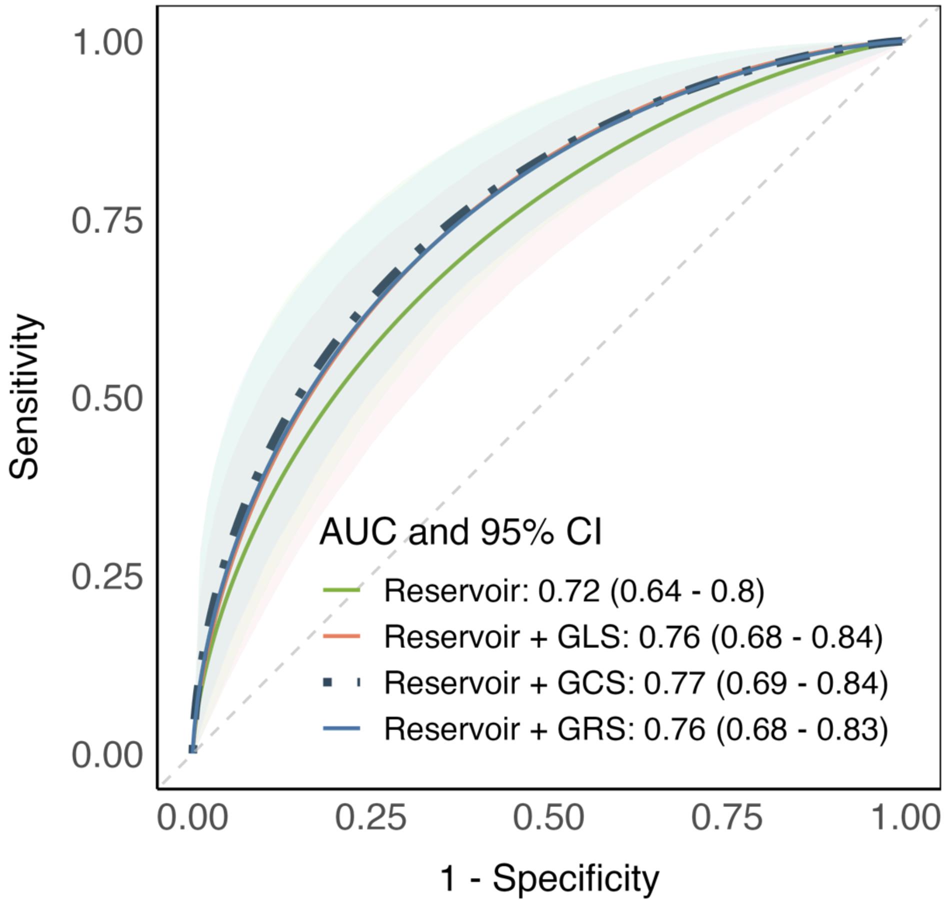

Supplemental Figure S2.

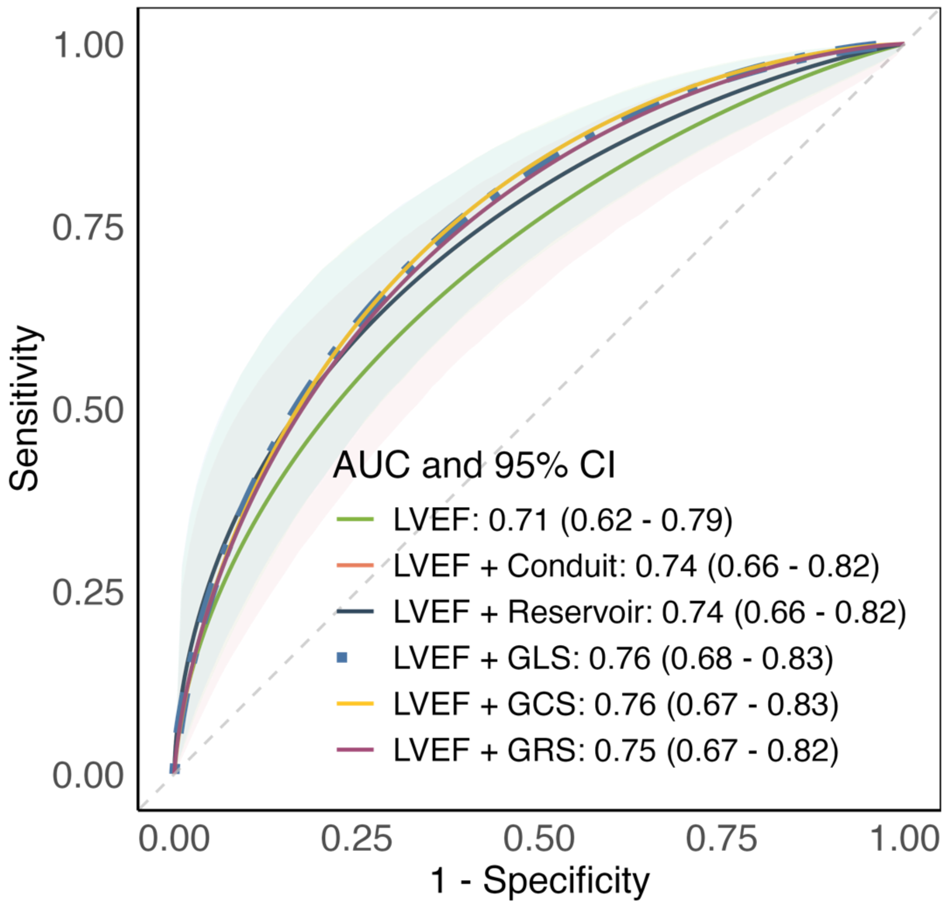

Supplemental Figure S3.
